# Supplementary material for: MetaRibo-Seq measures translation in microbiomes
Source: Nat Commun. 2020 Jun 29;11:3268. doi: 10.1038/s41467-020-17081-z (PMC7324362; doi:10.1038/s41467-020-17081-z)
Supplement: Supplementary file 10 — Supplementary Data 7 [file 41467_2020_17081_MOESM10_ESM.zip › File2/Confidence_High_Taxonomy/262751_out.krona.html]

Javascript must be enabled to view this page.

members
magnitude
magnitudeUnassigned
count
unassigned
taxon
rank

262751\_out

41

2
41
superkingdom

1224
41
phylum

41
28216
class

41
80840
order

41
995019
family

41

SRS015663\_contig\_number\_contig-100\_12125.133273SRS017433\_contig\_number\_14079SRS017701\_contig\_number\_14947SRS077641\_contig\_number\_6431SRS104912\_contig\_number\_contig-100\_2039.118482SRS1055076\_contig\_number\_contig-100\_8185.43888
6
40544
genus

species
6

SRS015264\_contig\_number\_contig-100\_1546.103891SRS019397\_contig\_number\_10761SRS054905\_contig\_number\_22026SRS055017\_contig\_number\_20412SRS098571\_contig\_number\_59099SRS147271\_contig\_number\_19234
40545

species
1897024

SRS017375\_contig\_number\_835SRS017821\_contig\_number\_26439SRS018888\_contig\_number\_9368SRS019808\_contig\_number\_20714SRS044535\_contig\_number\_21781SRS050026\_contig\_number\_4982SRS050299\_contig\_number\_14627SRS053649\_contig\_number\_16980SRS075021\_contig\_number\_6243SRS076756\_contig\_number\_13995SRS077086\_contig\_number\_10904SRS078665\_contig\_number\_4751SRS104036\_contig\_number\_7862SRS104311\_contig\_number\_21872SRS104912\_contig\_number\_14755SRS142923\_contig\_number\_22025SRS144506\_contig\_number\_43867SRS145497\_contig\_number\_17834SRS146764\_contig\_number\_22505SRS148424\_contig\_number\_34554
20


SRS048981\_contig\_number\_contig-100\_211.54560SRS054956\_contig\_number\_14381SRS076804\_contig\_number\_11892SRS101376\_contig\_number\_23073SRS1041112\_contig\_number\_210SRS1041134\_contig\_number\_contig-100\_10831.45928SRS105082\_contig\_number\_17113SRS143876\_contig\_number\_26925SRS147919\_contig\_number\_18907
1574262
9
species
